# Structure and variation of CRISPR and CRISPR-flanking regions in deleted-direct repeat region *Mycobacterium tuberculosis* complex strains

**DOI:** 10.1186/s12864-017-3560-6

**Published:** 2017-02-15

**Authors:** Paul Jeffrey Freidlin, Israel Nissan, Anna Luria, Drora Goldblatt, Lana Schaffer, Hasia Kaidar-Shwartz, Daniel Chemtob, Zeev Dveyrin, Steven Robert Head, Efrat Rorman

**Affiliations:** 1 National Mycobacterium Reference Center, National Public Health Laboratory Tel Aviv, Ministry of Health, Tel Aviv, Israel; 2 current address: Ben-Gurion University of the Negev, Beer Sheva, Israel; 3 Scripps Research Institute, San Diego, CA USA; 4 Department of Tuberculosis and AIDS, Ministry of Health, Jerusalem, Israel; 5 National Public Health Laboratory Tel Aviv, Ministry of Health, Tel Aviv, Israel

**Keywords:** *Mycobacterium tuberculosis* complex MTBC, Next Generation Sequencing NGS, CRISPR-Cas, Spacers, *cas1*, IS6110, Deleted-direct repeat region deleted-DR, Region of difference deletion RD deletion, Spoligotype, MIRU-VNTR

## Abstract

**Background:**

CRISPR and CRISPR-flanking genomic regions are important for molecular epidemiology of *Mycobacterium tuberculosis* complex (MTBC) strains, and potentially for adaptive immunity to phage and plasmid DNA, and endogenous roles in the bacterium. Genotyping in the Israel National Mycobacterium Reference Center Tel-Aviv of over 1500 MTBC strains from 2008–2013 showed three strains with validated negative 43-spacer spoligotypes, that is, with putatively deleted direct repeat regions (deleted-DR/CRISPR regions). Two isolates of each of three negative spoligotype MTBC (a total of 6 isolates) were subjected to Next Generation Sequencing (NGS). As positive controls, NGS was performed for three intact-DR isolates belonging to T3_Eth, the largest multiple-drug-resistant (MDR)-containing African-origin cluster in Israel. Other controls consisted of NGS reads and complete whole genome sequences from GenBank for 20 intact-DR MTBC and for 1 deleted-DR MTBC strain recognized as CAS by its defining RD deletion.

**Results:**

NGS reads from negative spoligotype MTBC mapped to reference H37Rv NC_000962.3 suggested that the DR/CRISPR regions were completely deleted except for retention of the middle IS6110 mobile element. Clonally specific deletion of CRISPR-flanking genes also was observed, including deletion of at least *cas2* and *cas1* genes. Genomic RD deletions defined lineages corresponding to the major spoligotype families Beijing, EAI, and Haarlem, consistent with 24 loci MIRU-VNTR profiles. Analysis of NGS reads, and analysis of contigs obtained by manual PCR confirmed that all 43 gold standard DR/CRISPR spacers were missing in the deleted-DR genomes.

**Conclusions:**

Although many negative spoligotype strains are recorded as spoligotype-international-type (SIT) 2669 in the SITVIT international database, this is the first time to our knowledge that it has been shown that negative spoligotype strains are found in at least 4 different 24 loci MIRU-VNTR and RD deletion families. We report for the first time negative spoligotype-associated total loss of CRISPR region spacers and repeats, with accompanying clonally specific loss of flanking genes, including at least CRISPR-associated genes *cas2* and *cas1*. Since *cas1* deleted *E.coli* shows increased sensitivity to DNA damage and impaired chromosomal segregation, we discussed the possibility of a similar phenotype in the deleted-DR strains and Beijing family strains as both lack the *cas1* gene.

**Electronic supplementary material:**

The online version of this article (doi:10.1186/s12864-017-3560-6) contains supplementary material, which is available to authorized users.

## Background

The National Public Health Laboratory Tel Aviv, National Mycobacterium Reference Center routinely genotypes all new culture-positive tuberculosis cases in Israel [1] with 43 spacers reverse line blot spacer oligonucleotide typing (spoligotyping) [2–4], and 24 loci mycobacterial interspersed repeat units – variable number tandem repeats (MIRU-VNTR) typing [5] using multiplex PCR and capillary electrophoresis [6, 7]. The results are stored in Excel (Microsoft), BioNumerics (Applied Maths, Belgium), and WHOnet databases. From 2008–2013, over 1500 strains were genotyped and tested for drug sensitivity to first line, and when necessary, second line drugs. Six drug-sensitive isolates from three different patients A, B, and C (two isolates per patient) yielded negative spoligotyping results and three different 24 loci MIRU-VNTR profiles respectively (Additional file 1: Table S1).

**Fig 1:**
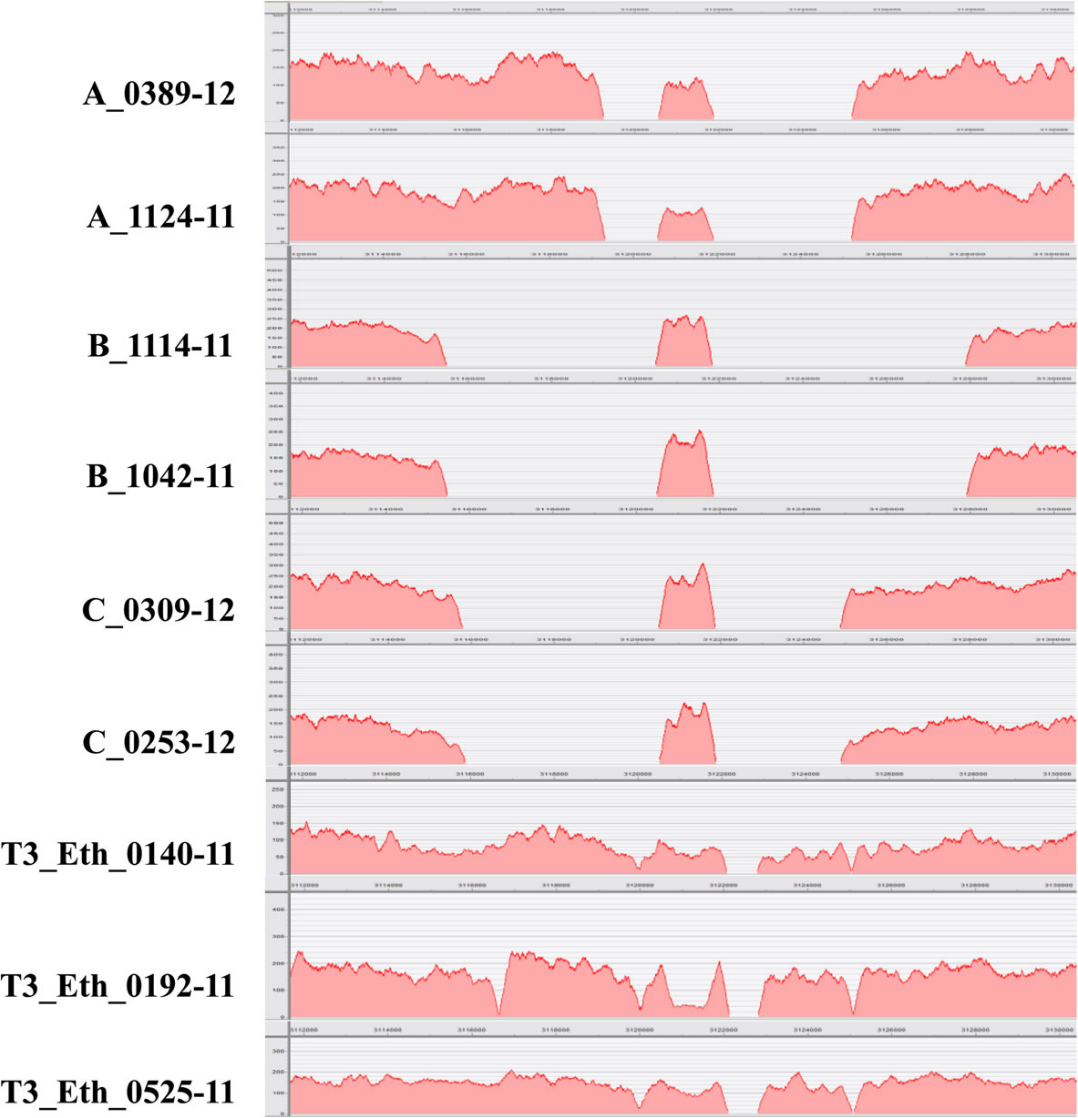
Location and coverage of H37Rv-mapped NGS-reads of negative-spoligotype strains A, B, C and intact-DR T3_Eth

**Fig 2:**
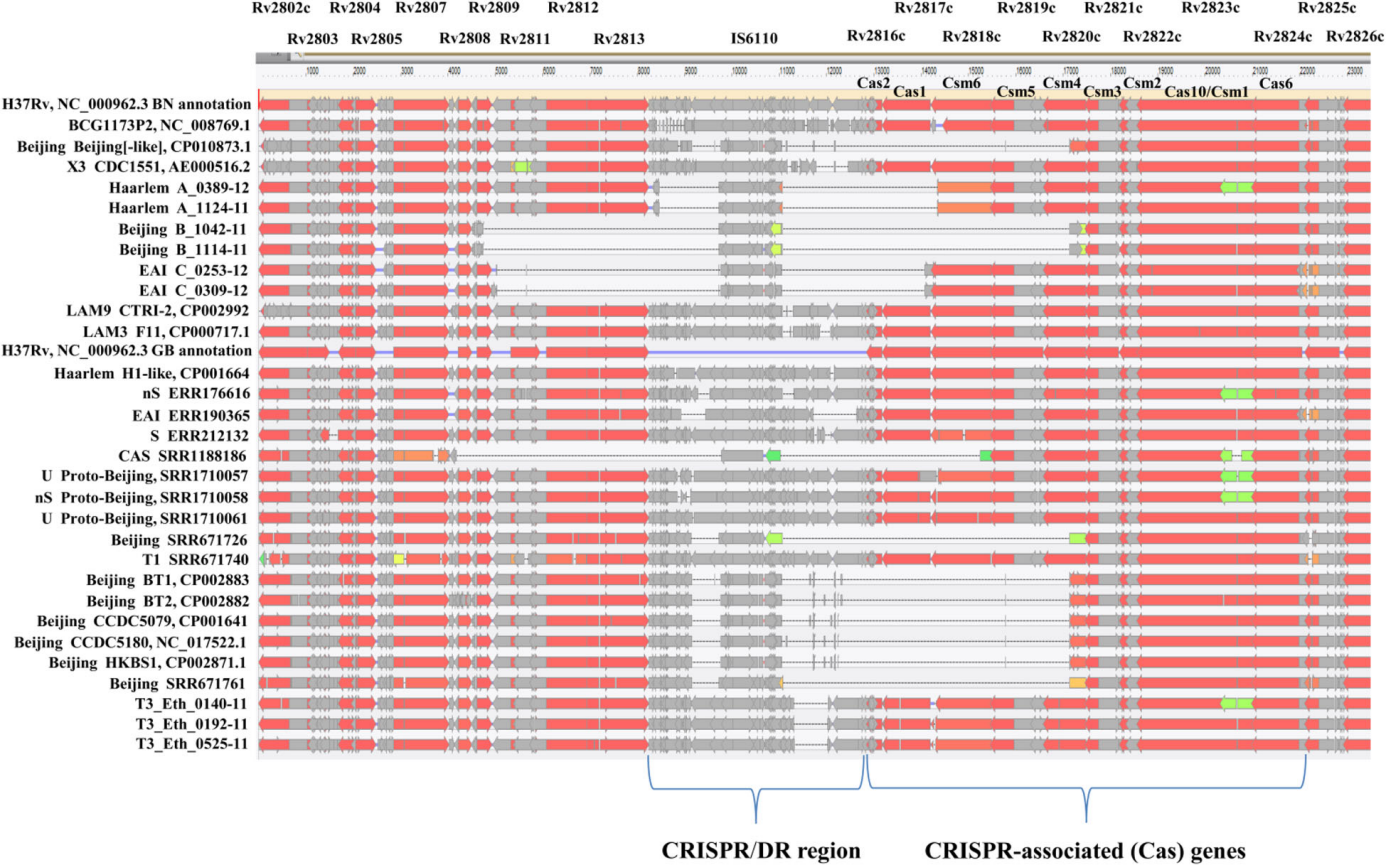
Structure and variation of CRISPR and CRISPR-flanking regions in deleted-DR and intact-DR MTBC

Variation in the structure of CRISPR and CRISPR-flanking regions of these negative spoligotype MTBC isolates was observed when NGS reads were mapped on reference whole genome sequence H37Rv NC_000962.3. This yielded visualization of coverage (Fig. 1). After substitution of manual-PCR-derived DR-spanning sequences for the corresponding map-derived regions, further details of structural variation were revealed by alignments of annotated subsequences containing CRISPR plus CRISPR-flanking regions to the annotated reference H37Rv sequence (Fig. 2).

In addition to the 6 drug-sensitive deleted-DR isolates, two drug sensitive and 1 multiple-drug-resistant (MDR) intact-DR strains from T3_Eth (SIT 149), our largest African-origin MDR-containing cluster, were analyzed by NGS (Table 1, Additional file 1: Table S1, Additional file 2: List S1). These provided positive controls for the sensitivity of our methods to correctly characterize from NGS data, the CRISPR-Cas regions of intact-DR MTBC, especially with respect to the presence or absence of CRISPR spacers.

**Table 1:** Characteristics of NGS sequenced deleted-DR sample strains A, B, C and intact-DR T3_Eth, and whole genome sequences obtained on-line or assembled from on-line read sets#

MTBC Strain, ID	Spoligotype Octal	SIT or RD Family	Drug Res	CRISPR IS6110	mutation in			regions of difference (RD)							
					*mutT4*	*T2*	*ogt*	105	207	181	150	142	182	239	750
Beijing															
BT1, CP002883	000000000003771	1,Beijing	TDR^a^	inv	48	58	12	del	del	del	wt	wt	wt	wt	wt
BT2, CP002882	000000000003771	1,Beijing	TDR^a^	inv	48	58	12	del	del	del	wt	wt	wt	wt	wt
CCDC5079, CP001641	000000000003771	1,Beijing	DS^b^	inv	48	58	12	del	del	del	wt	wt	wt	wt	wt
CCDC5180, NC_017522.1	000000000003771	1,Beijing	MDR^b^	inv	48	58	12	del	del	del	wt	wt	wt	wt	wt
HKBS1, CP002871.1	000000000003771	1,Beijing	DS^a^	inv	48	58	12	del	del	del	wt	wt	wt	wt	wt
Beijing [−like], CP010873.1	000000000003731	190,Beijing	MDR^c^	inv	48	58	12	del	del	del	wt	wt	wt	wt	wt
SRR671761	000000000003771	1,Beijing	?^d^	unk	48	58	12	del	del	del	del	wt	wt	wt	wt
SRR671726	000000000003770	1,Beijing	XDR^d^	unk	48	58	12	del	del	del	wt	wt	wt	wt	wt
B_1042-11	000000000000000	nS,Beijing	DS^e^	wt	48	58	12	del	ext	del	wt	wt	wt	wt	wt
B_1114-11	000000000000000	nS,Beijing	DS^e^	wt	48	58	12	del	ext	del	wt	wt	wt	wt	wt
Proto-Beijing															
SRR1710057	777777777777731	246, U	panS^d^	unk	wt	wt	wt	ext	wt	wt	wt	wt	wt	wt	wt
SRR1710058	777777777775331	nS	panS^d^	unk	wt	wt	wt	ext	wt	wt	wt	wt	wt	wt	wt
SRR1710061	777777777777771	523, U	panS^d^	unk	wt	wt	wt	ext	wt	wt	wt	wt	wt	wt	wt
Non-Beijing															
ERR176616	777777607060771	nS	MDR^d^	unk	wt	wt	wt	wt	wt	wt	wt	wt	wt	wt	wt
ERR190365	000037777403471	nS,EAI	DR^d^	unk	wt	wt	wt	wt	wt	wt	wt	wt	wt	del	wt
ERR212132	776337777760771	71, S	panS^d^	unk	wt	wt	wt	wt	wt	wt	wt	wt	wt	wt	wt
SRR671740	777777777760771	53, T1	XDR^d^	unk	wt	wt	wt	wt	wt	wt	wt	wt	wt	wt	wt
SRR1188186	000000000000000	nS,CAS	panS^d^	unk	wt	wt	wt	wt	new	wt	wt	wt	wt	wt	del
A_0389-12	000000000000000	nS,Haarlem	DS^e^	wt	wt	wt	15	wt	new	wt	wt	wt	del	wt	wt
A_1124-11	000000000000000	nS,Haarlem	DS^e^	wt	wt	wt	15	wt	new	wt	wt	wt	del	wt	wt
C_0253-12	000000000000000	nS,EAI	DS^e^	inv	wt	wt	wt	wt	new	wt	wt	wt	wt	del	wt
C_0309-12	000000000000000	nS,EAI	DS^e^	inv	wt	wt	wt	wt	new	wt	wt	wt	wt	del	wt
T3_Eth_0140-11	777000377760771	149,T3_Eth	DS^e^	unk	wt	wt	wt	wt	wt	wt	wt	wt	wt	wt	wt
T3_Eth_0192-11	777000377760771	149,T3_Eth	MDR^e^	unk	wt	wt	wt	wt	wt	wt	wt	wt	wt	wt	wt
T3_Eth_0525-11	777000377760771	149,T3_Eth	DS^e^	unk	wt	wt	wt	wt	wt	wt	wt	wt	wt	wt	wt
BCG1173P2, NC_008769.1	676773777777600	482,BCG	DR^f^	wt	wt	wt	wt	wt	wt	wt	wt	del	wt	wt	wt
CDC1551, AE000516.2	700076757760771	549,X3	DS^g^	wt	wt	wt	wt	wt	wt	wt	wt	wt	wt	wt	wt
CTRI-2, CP002992	777777607760771	42,LAM9	DS^g^	wt	wt	wt	wt	wt	wt	wt	wt	wt	wt	wt	wt
F11, CP000717.1	776177607760771	33,LAM3	DS^g^	wt	wt	wt	wt	wt	wt	wt	wt	wt	wt	wt	wt
Haarlem, CP001664	775777774020751	H1-like	DR^g^	wt	wt	wt	15	wt	wt	wt	wt	wt	del	wt	wt

^#^ID, sample, read set or NCBI accession number; octal, from spoligosome17, SIT, spoligotype international type and associated family; nS, no SIT; Drug Res, resistant to drug, I-isoniazide, R-rifampicin, E- ethanbutol, S-streptomycin, P-pyrazinamide, DS (drug sensitive): none, DR (drug resistant): at least one but not I&R together: MDR (multiple drug resistant): at least I&R, XDR (extensively drug resistant): at least MDR plus defined 2^nd^ line drugs consisting of at least 1 quinolone and at least one of the injectable drugs kanamycin, capreomycin or amikacin, TDR (totally drug resistant): resistant to all 1^st^ and 2^nd^ line drugs. a-g: Additional file 2: List S1 – sources of drug sensitivity data; wt, identical to reference genome H37Rv NC_000962.3; CRISPR IS6110, orientation of the IS6110 mobile element found in the direct repeat region, wt: same orientation as in H37Rv, inv: inverted, unk: unknown; mutation in *mutT4* (Rv3908), codon 48: Arg CGG GGG Gly; mutation in *mutT2* (Rv1160), codon 58: Gly GGA CGA Arg; mutation in *ogt* (Rv1316c), silent codon 12: Gly CCC TCC Gly, missense codon 15: Thr GGT GCT Ser (note: ogt gene is in inverse orientation); regions of difference RD, 105: proto and all modern Beijing, 207: LSP in CRISPR region defining modern Beijing – includes *cas* gene deletions, 181-150-142: polymorphic LSPs for Beijing, 182: marker for Haarlem, 239: marker for EAI, 750: marker for CAS

Complete loss of all Direct Repeat region (DR) repeats and spacers, negative spoligotyping results, and loss of genes in flanking regions has been reported previously for MTBC with negative spoligotypes [8, 9] identical to those currently assigned to SIT 2669 [10] and for other bacteria [11], however, the molecular structures of the CRISPR regions (the newer name for DR) and flanking CRISPR-associated (Cas) regions [12] were not reported, except for a comparison to the homologous genomic region in non-tuberculous mycobacteria (NTM) [13]. The evidence reported herein clearly showed that the deleted-DR and accompanying deleted CRISPR-flanking genes of the MTBC described in this study were not an instance of the ORFs absent in NTM species (Fig. 2). Also, this study is the first known to us to employ identification of Region of Difference (RD) deletions in order to assign negative spoligotype MTBC to their corresponding major spoligotype families.

The MTBC RD deletions are genomic long sequence polymorphisms (LSPs) [14, 15] which were detected in this study by aligning *Mycobacterium tuberculosis* complete genome sequences (obtained from public on-line databases or derived from NGS reads mapped on the H37Rv genome, Table 1) with the MTBC reference strain H37Rv complete genome sequence [16]. The MTBC RD deletions, and also an extensive set of single nucleotide polymorphisms (SNPs), have been shown by Coll et al. [17] in an on-line available application for analysis of NGS reads, to have phylogenetic behavior that corresponds well with the phylogenetic distribution obtained by analysis of 43-spacer spoligotypes. 43-spacer spoligotypes have been assigned type numbers and family names [10, 18]. The 43-spacer spoligotypes are more informative than the RD types and SNP types, thus any given specific 43-spacer spoligotype cannot be inferred from an RD type or SNP type, but the spoligotype family can be inferred from RD or SNP information, and in addition some sub-lineage information can be obtained from the SNP type [17]. Therefore, when one encounters a negative spoligotype by conventional screening for the 43 CRISPR spacers, if NGS sequencing can be performed for the deleted-DR strain, two convenient ways exist to determine the spoligotype family: 1) map the NGS reads to reference H37Rv, align the constructed MTBC sequence with the H37Rv sequence, and search for the relevant RD deletions, and 2) submit the NGS reads to the Coll et al. [17] site for analysis of RD deletions and SNPs. The RD data shown in Table 1 suggested that a third way could be developed which would not require sequencing for NGS reads: a possible multiplex PCR probe for relevant RD deletions, which could be analyzed by fragment analysis as in MIRU-VNTR typing, or by real time PCR. Various methods have been proposed for spoligotyping from NGS reads [19–22], but all involve detection of CRISPR spacers and thus would only yield negative results with our deleted-DR negative spoligotype strains.

A combination of the PCR-based methods 43 spacer spoligotyping and 24 loci MIRU-VNTR typing has replaced RFLP-typing as the MTBC genotyping screen of choice, due to the speed and convenience of the PCR-based methods, genotype discrimination as good as or better than that for RFLP-typing, and the ability to store the PCR-based results in easily manipulated digital format [5]. On a population level, screening for spoligotype family provides insight into the bio-geographic burden of TB in the world and for any particular country [1, 10, 18]. Thus it is useful to be able to determine the spoligotype family for a strain that has a negative spoligotype by conventional screening. While in general the more informative 24 loci MIRU-VNTR type will still phylogenetic-wise segregate according to its complementary spoligotype [5], for certain MIRU-VNTR profiles the miru-spoligo correlation may be difficult to establish. A published example of this [7] showed that the MIRU-VNTR profiles in spoligotype families CAS and Beijing can sometimes be difficult to distinguish. In another case, very similar MIRU-VNTR profiles were found in both the T and LAM spoligotype families (unpublished). In these cases it was not only useful, but indeed necessary, to determine at least the spoligotype family. On a clinical level, the combination of spoligotype and MIRU-VNTR type complements and guides classical epidemiological investigation to clarify questions of transmission between individuals, transmission chains among larger groups of individuals, questions about whether a recurrence of TB is due to reactivation or reinfection, and resolution of questions arising when a lab provides a positive TB report that is inconsistent with the physician's observation – that is- the possibility of a false positive due to lab contamination. Often 24 loci MIRU-VNTR alone supplementing classical epidemiology is enough to resolve these issues. Nevertheless, if the strains involved have negative spoligotypes on conventional screening, knowledge of at least the spoligotype family (and even better, the sub-lineage from SNP data) can help validate the results concluded from classical epidemiology supplemented with MIRU-VNTR typing. Finally, there are often gaps in epidemiological knowledge when trying to establish transmission chains among larger groups of individuals. These knowledge gaps can be exacerbated by subtle polymorphisms (1 locus, 1 copy number) in MIRU-VNTR profile, where knowing only the spoligotype *family* will not be enough to firmly establish the transmission chain. Therefore, when confronted with negative spoligotypes in large putative transmission chains, it seems reasonable to recommend that all the strains should be processed for NGS reads. Then the NGS reads for the negative spoligotype strains should be used for spoligotype family determination [17], and subsequently for SNP clonality-check comparisons among the strains to delineate the outbreak [23, 24].

*Mycobacterium tuberculosis* complex (MTBC) bacteria possess a Type III-A CRISPR-Cas system structurally homologous to Type III-A of other genera [12]. Active Type III-A systems provide adaptive immunity to bacteriophage and plasmids [12, 25, 26], and perform endogenous roles, for example, endogenous DNA repair via Cas1 endonuclease [27].

In MTBC the CRISPR region is also known as the direct repeat region (DR). Polymorphism in the number and type of 43 spacers in the DR/CRISPR region of any given MTBC strain, has been and continues to be extensively used for molecular epidemiology of MTBC, especially for overviews of phylogenetic and bio-geographical distributions of clades [2, 4, 10, 18]. Fourty three-spacer-SPO-plus-24-loci-MIRU genotyping provides molecular epidemiology at the resolution of clonal transmission [5], with the exception of certain instances, for example, of convergent evolution [28].

The functional activity of the MTBC CRISPR-Cas system remains to be investigated, but the highly conserved sequence and order of the 43 CRISPR/DR region spacers [3] which is the basis for the epidemiological and phylogenetic usefulness of polymorphism on spacer oligonucleotide typing (spoligotyping), has been interpreted to imply at least partial inactivity of the MTBC CRISPR-Cas system [29]. This interpretation presumes that the insertion of new spacers in the CRISPR/DR region is one of the fundamentals of, and evidence for, adaptive immunity, and thus lack of evidence for insertion of new spacers in MTBC implies at least partial inactivation of the CRISPR-Cas adaptive immune system in MTBC. The MTBC Type III-A *CRISPR-Cas* system [12] is defined by the presence of *cas10/csm1* and *csm* accessory genes, and is predicted to be in some manner active on the basis of the presence of intact *cas1* and *cas2* genes. In addition, some sort of activity even if only in endogenous roles, can be predicted from GenBank annotation that informs of mass spectroscopy support [30] for the existence of most of the *cas* gene products, including Cas1in the reference MTBC strain H37Rv. The lineage-defining RD 207 deletion [14, 31] of the super-spreader MDR-containing Beijing family includes complete deletion of *cas1* and *cas2* genes (Fig. 2), so possession of a fully active Type III-A CRISPR-Cas system is not a prerequisite for a successful (with respect to pathogenicity, transmissibility, and adaptability) MTBC lineage. Interestingly, the complete loss of CRISPR spacers in *E. coli* gave the same DNA-repair deficient phenotype as deletion of the *E. coli cas1* (*ygbT*) gene [27]. Using *M. smegmatis* as a model for MTBC [32], Cas2 [33] by itself was shown to be deleterious to *Mycobacterium* survival, thus it may be significant that deletion of *cas1* was always accompanied by deletion of at least *cas2*.

In summary, the results showed that complete deletion of DR region repeats and spacers in negative-spoligotype MTBC was accompanied by clonal-specific loss of flanking genes, always accompanied by complete deletion of *cas2*, complete deletion of putative (functional activity still unknown in MTBC) DNA-repair gene *cas1*, and retention of the middle IS6110 mobile element. For the first time it was shown that negative spoligotype MTBC nevertheless could be assigned to major spoligotype families by identification of NGS-revealed corresponding lineage-defining RD deletions. Since there was only 1 deleted-DR strain-type (based on at most two isolates) per RD-defined family, there was not enough information to decide if the loss of *cas* genes was solely clonally specific, or perhaps had lineage-defining features.

## Methods

### Growth of *Mycobacterium tuberculosis* strains, and drug sensitivity tests

All approximately 1500 new culture-positive MTBC strains collected during 2008–2013 were analyzed. Cultures were grown and identified as MTBC as previously described [7]. Drug sensitivity tests were performed as previously described [7].

### Extraction of MTBC DNA

Preparations of RFLP-grade MTBC DNA for NGS sequencing, and of “crude” sonicated, heated extract for genotyping, were done as previously described [7].

### Genotyping of MTBC DNA

Routine genotyping of MTBC DNA by 43 spacer spoligotyping (reverse line blot kit, Omicum, India) and 24 loci MIRU-VNTR typing (in house multiplex PCR and amplicon-sizing by capillary electrophoresis) were done as previously described [6, 7]. BCGP2 was the generous gift of Professor Hillel Bercovier, Hebrew University of Jerusalem. European Communicable Disease Center Proficiency Test (ECDCPT) samples 21 and 25, and ECDCPT H37Rv control DNA were obtained originally as proficiency test materials [34]. Genotyping results were stored in an Excel (Microsoft) database, a BioNumerics ver7.6 database, and a WHONET database.

### NGS Assay and bioinformatics analyses at the Scripps Research Institute, including 1) coverage-curves illustrating deletion of CRISPR/DR-Cas regions present in the template reference H37Rv chromosome sequence, and 2) determination of spoligotypes from NGS reads

RFLP-grade DNA samples (micrograms of highly concentrated, highly purified, high quality DNA) were sent to Mgr. Steven Robert Head and Mgr. Lana Schaffer of the Scripps Research Institute in San Diego, California USA for NGS and draft bioinformatics analysis.

One microgram of each sample was sheared on the Covaris S2. Each sample was then end repaired, a-tailed and adapter ligated. The samples were cleaned with Agencourt beads and treated with 6 cycles of PCR. After PCR the samples were cleaned using Zymo 25 columns and loaded on a 2% agarose gel. The gel was cut from 400–700 bp and cleaned again with the Zymo25 column.

The samples were sequenced on an Illumina Miseq system as paired-end reads each with length 300 bp. The alignment was performed using Novoalign v3.00.05 (http://www.novocraft.com) without soft clipping to allow reads which cross the ends of the reference sequence as would be the case of a circular genome. The *Mycobacterium tuberculosis* reference sequence used for the alignment was MTB-H37Rv-asm19595v2 and for repeat sequences not in the mapped-to-H37Rv MTBC sequence was *Mycobacterium bovis* AF2122/97. The reads were preprocessed by trimming adaptor sequences (GATCGGAAGAGCACACGTCTG), trimming by base call quality scores lower than 26, removing unmapped reads and duplicate reads, and removal of reads with mapping quality score less than one. Repeat sequence coordinates and sequences were taken from the Jansen paper [35].

For coverage curves illustrating deletion of CRISPR-DR-Cas regions that were present in the template H37Rv chromosome sequence, the aligned bam files were converted to wig files using bamtools, bedtools, and ucsc-tools. The wig files were imported into UCSC Microbial Genome Viewer (http://microbes.ucsc.edu).

Recovery of spoligotypes from NGS data was accomplished as follows. A bed file was created using the coordinates of the 43 DR repeat units. This bedfile and the aligned bam files were used with “samtools bedcov” tool to output number of counts for each repeat unit. In order to produce a binary table matching the spoligotyping regions with less than 500 counts were considered absent.

### Bioinformatics at the National Public Health Laboratory Tel Aviv

**Analyses of our NGS raw data** were accomplished by use of BioNumerics version 7.5 and 7.6 (Applied Maths, Belgium) import and processing of 1) cleaned paired-end and single-end fastq reads received from Scripps, 2) reads formatted as sra (in fastq format) and fastq files downloaded from NCBI or other sources, and 3) fastq or EMBO/GB format reference whole chromosome sequences or contributed chromosome sequences from NCBI or other sources. For visualization of the molecular details of the CRISPR-DR and flanking regions (including Cas genes), reads were resequenced/mapped onto reference H37Rv NC_000962.3 using default parameters with the following changes: accept paired-end reads, minimum coverage 10, minimum sequence length before considered for contig 500 nt. The “coverage” was examined as percentage of the H37Rv genome matched, and as the number of reads contributing to each base pair assignment.

The mapped chromosomes were annotated with H37Rv GenBank and BioNumerics-predicted (based on possible reading frames) features and matrix compared and aligned in chromosome comparison mode using default parameters. For decisions about reading frames and codon assignments, reference H37Rv NC_000962.3 annotated only with GenBank annotation was included in comparisons.

Default parameters were: **I**. *Comparison Project type*: full sequence based; Matrix: matrix; self-comparison of sequences; direct sequence orientation; inverted sequence orientation; Seed extension DNA seed: minimum 15 matches in window size of 25 bases; Amino acid seed: minimum 6 matches in window size of 10 amino acids; Stretch extension Minimum 15 matches in window size of 30 bases; Minimal stretch length: 50; **II**. *Seeds Seed database*: seed 11111, type: DNA, length:5; Seeds used in project: seed 11111, type: Transl, length:5, and **III**. *Alignment settings* template entry: usually the GB plus BN annotated reference H37Rv; stretch import minimum length: 0; Minimum identity: 0; Stretch mapping Open gap penalty: 10; Extend gap penalty 10; do NOT allow repetition of mapped stretches (box should NOT be checked); full alignment of stretch ends: checked; Full alignment settings: open gap penalty (%): 100; extend gap penalty (1%): 0; use fast algoritm: checked; K-tuple size:2; No. of diagonals (1–200): 99. This generated a schematic suitable for visualization of deletions, while choosing “show text view” while in alignment sub-mode in the comparison mode allowed visualization of single nucleotide polymorphisms (SNPs). An important point is that any given set of parameters has its own strengths and limitations. The default parameters of BioNumerics 7.5 and 7.6 Chromosome Comparison did not detect inverse IS6110 in the CRISPR-Cas region (it appeared as a deletion), nor were they suitable for comparison of shorter sequences (for example, sequences of 44 thousand nucleotides/nt or less).

The region containing the CRISPR-Cas sequence plus flanking regions was cut out (maximum approximately 44 thousand nt), annotated and used for further comparisons in the alignment window when high similarity was expected, or in the chromosome comparison mode and alignment sub-mode with appropriate parameters when some dissimilarity was expected.

**The appropriate parameters for comparison of shorter sequences in the Chromosome Comparison mode** were (*only parameters differing from the default parameters detailed above are given*): **I**. *Comparison Seed extension*: DNA seed: minimum 6 matches in window size of 15 bases; Amino acid seed: minimum two matches in window size of 5 amino acids; Stretch extension: minimum 6 matches in window size of 10 bases; Minimal stretch length: 12; **II**. *Seeds seed database*: seed: 111; type: DNA; length: 3; Seeds used in project: seed: 111; type: DNA; length: 3; **III**. *Alignment settings*: Full alignment settings: open gap penalty (%): 0; No. of diagonals (1–200): 30. Other parameter sets can be better for other uses, and must be determined empirically.

In order to obtain contig-corrected sequences for the six approximately 44 Kbp CRISPR plus CRISPR-flanking subsequences of the deleted-CRISPR strains from patients A, B, and C, the NGS-obtained H37Rv-mapped subsequences containing the CRISPR-spacers regions (the DR regions) were excised from the assembled mapped whole genome sequence at predetermined flanking genes on each side, and replaced with sequences of PCR validated contigs spanning the CRISPR-spacers-DR region, that is, with 5’ and 3’ ends of the contigs overlapping the conserved CRISPR-flanking genes (Fig. 2) characteristic for each deleted-DR strain. These “DRplus” sequences corrected for the appropriate contigs are found in Additional file 3: Sequences S1–S6. The CRISPR-flanking genes and their locations are listed in Additional file 4: Table S2.

**The PCR validated contigs** were constructed using primers inferred from the NGS data from mapped to H37Rv, and *de novo*, sequences (*de novo* sequences obtained using the open-source short-read assembly program “Ray” employed by the Power Assembler tool of Applied Mathematics software BioNumerics version 7.5 and version 7.6) according to which regions could be expected to be “conserved” in the CRISPR-spacers region itself (that is, the IS6110 mobile element), and in the immediately adjacent CRISPR-flanking regions characteristic for each deleted-DR strain. PCR and agarose gel electrophoresis were performed as previously described [7] and the PCR product purified on QIAQUICK (QIAGEN) columns. The purified PCR product was sent for forward and reverse sequencing to a commercial sequencing facility (HyLabs, Rehovot), and the sequences from the ab1 sequencing files were trimmed and used to construct contigs spanning the CRISPR-spacers region (BioNumerics version 7.5). Validation of sequence trimming and alignment for contig construction was done in parallel using a different bioinformatics tool (Clone Manager 9).

The H37Rv NC_000962.3 whole genome sequence locations, corresponding to the DR plus flanking regions (sequences named DRplus) of the mapped deleted-DR strains A, B, and C, were used for alignments for primer construction (Additional file 4: Table S2). The major primers used for manual PCR were as shown in Additional file 5: Table S3.

**Recovery of spoligotypes** from NGS reads, or from downloaded reference or contributed chromosomes, was accomplished by constructing a new BioNumerics version 7.5 (now 7.6) application, manuscript in preparation. In addition, the sequences for the DR region-spanning contigs for deleted-DR strains A, B, and C were examined by NCBI BLAST for sequences corresponding to spacer sequences.

**Drug sensitivity and lineage information** for downloaded reads was obtained either from the literature (Table 1, Additional file 2: List S1) [14, 15] or by uploading the reads to TB Profiler [36] which reported back the drug sensitivities for first and second line drugs, and projected lineages. RD deletion confirmation of lineages (Table 1) was obtained from BioNumerics Chromosome Comparison alignments of complete genome sequences (downloaded whole, or derived from assembly of H37Rv-mapped-reads) to the reference H37Rv sequence.

## Results and Discussion

### Variation in the CRISPR and CRISPR-flanking regions: clonal specific deletion of CRISPR-flanking regions in deleted-DR strains – including deletion of at least *cas1* and *cas2*, and retention of the IS6110 mobile element in deleted-DR strains

In Fig. 1, coverage of the homologous reference H37Rv sequences was approximately 150 reads for each isolate, with the negative spoligotype isolates A, B, and C showing wide gaps to the left and right of the middle sequence. The middle read-coverage length was suggestive of that of an IS6110 mobile element. The coverage of the template H37Rv genome (NC_000962.3) (4411532 bp) ranged from 97.4 to 99.1% for the deleted-CRISPR-spacers-Cas strains A, B, and C, and 98.3 to 99.2% for the T3_Eth strains.

Variations in the CRISPR and CRISPR-flanking regions of negative spoligotype (Additional file 1: Table S1) MTBC isolates from patients A, B, and C were observed in the visualization of NGS reads mapped on reference H37Rv NC_000962.3 (Fig. 1) and annotated alignments of DRplus (DR plus flanking sequences) subsequences to the DRplus reference H37Rv subsequence (Fig. 2). For comparison, intact-DR results from three NGS-sequenced T3_Eth were shown.

*DRplus subsequences* from isolates A, B, C, and isolate SRR1188186 which was assembled from downloaded reads, were aligned to reference template H37Rv subsequence (Fig. 2, Additional file 4: Table S2). A, B, and C – specific (that is, clonally specific) deletions of CRISPR-flanking genes were observed, including deletions of *cas* genes to the right of the retained mid IS6110. All 4 deleted-DR strains, retained their mid IS6110 mobile element, and deleted at least *cas2* and *cas1* (Fig. 2, Additional file 4: Table S2).

*The deleted-DR B isolates* showed a right-side deletion of the same size and location as that shown in Beijing-family strains (Fig. 2). Subsequent RD deletion identification of deleted-DR B isolates as Beijing (Table 1), suggested that the B deletion may be an extension of the Beijing lineage-defining RD207 deletion. This was further suggested by the fact that the deleted-DR B isolates shared the Beijing-specific mutator phenotype SNPs *mutT4*|*T2*|*ogt* [37] (Table 1). Thus we propose to name the deletion seen in the deleted-DR B isolates as “extended RD207” in accordance with the terminology adopted by Luo et al. [31] for the RD105 deletion in proto-Beijing: extended RD105 (Table 1). It was interesting, but of as yet unproven important significance as discussed below, that in contrast to the inverse orientation of the IS6110 mobile element in the DR region of many if not all validated Beijing-family sequences (Additional file 6, Figure S1) the orientation of IS6110 in the Beijing deleted-DR B isolates was wild type, that is, the same as for reference template H37Rv (Additional file 6: Figure S1, Table 1).

### Validation of retention of mid-IS6110 mobile element, and of loss of varying amounts of genomic regions flanking the deleted CRISPR/DR regions of isolates A, B, and C

In order to validate retention of IS6110, and clonally specific loss of flanking genomic sequences in deleted-DR isolates A, B, and C, primers were chosen from conserved flanking genes and the mid IS6110, and used to generate amplicons by manual PCR which were assembled into contigs for a DR-spanning sequence for each isolate A, B, and C. These validated DR-spanning sequences were substituted into the DRplus sequences of H37Rv-mapped isolates A, B, and C, resulting in a “corrected’ DRplus sequence for each isolate A, B, and C (Additional file 3: Sequences S1–6). Alignment of the corrected DRplus sequences to template H37Rv (Fig. 2) confirmed that mid-IS6110 was retained in each deleted-DR isolate A, B, and C. It was interesting that the orientation of this IS6110 of each Beijing deleted-DR isolate B was wild type with respect to H37Rv, and inverse with respect to the orientation of IS6110 observed in reference whole genome Beijing sequences (Additional file 6: Figure S1, Table 1). Alignment of the manual PCR corrected DR plus flanking sequences to template H37Rv (Fig. 2) also confirmed the clonally specific loss of CRISPR-flanking regions, as shown in Fig. 2 and detailed in Additional file 4: Table S2. Each deleted-DR isolate A, B, and C showed deletion of at least *cas2* and *cas1* from among the *cas* genes (Fig. 2, Table 2, Additional file 4: Table S2).

**Table 2:** The presence or absence of mutations in CRISPR-associated genes in *Mycobacterium tuberculosis* complex strains compared to reference template H37Rv NC_000962.3 4411532 bp. The mutations are listed in ascending order of their location relative to the reference H37Rv chromosome

Type	Gene	Location	SNP	Change	Description	Rv
deletion	*cas2*	3123625			Beijing RD207 & all deleted-CRISPR strains	Rv2816c
deletion	*cas1*	3123967			Beijing RD207 & all deleted-CRISPR strains	Rv2817c
SNP	*cas1*	3124014	cga/cgc	Ser/S->Ala/A	T3_Eth defining	Rv2817c
SNP	*cas1*	3124352	gct/gat	Ser/S->Ile/I	T3_Eth defining	Rv2817c
SNP	*cas1*	3124743	cac/caa	Val/V->Leu/L	proto-Beijing defining	Rv2817c
deletion	*csm6*	3124996			Beijing RD207 & deleted-DR A (Haarlem)	Rv2818c
SSP	*csm6*	3125070			T3_Eth specific	Rv2818c
SSP&SNPs	*csm6*	3125120			proto-Beijing specific	Rv2818c
SNP	*csm6*	3125171	gtt/gct	Asn/N- > Ser/S	ERR212132 specific	Rv2818c
SSP	*csm6*	3125690			ERR212132 specific	Rv2818c
SNP	*csm6*	3125235	ttg/tta	Gln/Q- > stop	BCG specific	Rv2818c
deletion	*csm5*	3126240			Beijing RD207	Rv2819c
SNP	*csm5*	3126279	atc/att	Asp/d- > Asn/N	SRR671740 specific	Rv2819c
SNP	*csm5*	3127273	gtg/gtt	His/H- > Asn/N	proto-Beijing defining	Rv2819c
SNP	*csm4*	3127466	tcg/gcg	Arg/R- > Arg/R	BCG specific	Rv2820c
SNP	*csm4*	3127717	tgt/tgc	Thr/T- > Ala/A	T3_Eth defining	Rv2820c
truncation	*csm4*	3127930			Beijing RD207 truncation	Rv2820c
SNP	*csm4*	3127931	ttt/att	Lys/K- > Asn/N	Beijing-defining	Rv2820c
None^a^	*csm3*					Rv2821c
None^a^	*csm2*					Rv2822c
SNP	*cas10/csm1*	3129359	tcc/tct	Gly/G- > Arg/R	CDC1551 specific	Rv2823c
SNP	*cas10/csm1*	3129675	gcg/gtg	Arg/R- > His/H	C_0253-12 and C_0309-12 specific	Rv2823c
SNP	*cas10/csm1*	3130682	gcc/acc	Gly/G- > Gly/G	F11 specific	Rv2823c
SNP	*cas10/csm1*	3131188	gag/gaa	Leu/L- > Phe/F	Beijing BT2 specific	Rv2823c
SSPs & SNPs	*cas10/csm1*	3131470			Found in all MTBC strains	Rv2823c
None^a^	*cas6*					Rv2824c

^a^None: no mutations listed for the strains examined; Location: base pair of H37Rv NC_000962.3 where SNP can be found, or base pair approximately in the center of a given deletion or insertion; *SSP* short sequence polymorphism, *SNP* single nucleotide polymorphism

The absence of mutations in *csm3*, *csm2*, and *cas6* (Table 2) could mean that those genes cannot tolerate mutations due to the importance of their sequence, or transcription or translation products. However, mutations could appear if a larger set of strains were examined, a possibility suggested by the recent report that the *cas6* gene is dispensable in Type III CRISPR-cas systems [12].

### Did the retained IS6110 mobile element have a role in generating the CRISPR and flanking-sequences deletions observed in the deleted-DR strains?

The mobile element IS6110 inserts into the *Mycobacterium tuberculosis* genome in a variety of ways with a variety of potential effects [38], some of which we have discussed previously [7]. The DR/CRISPR region is a hot spot for IS6110 insertions, where there can be 0, 1, or more IS6110 elements inserted in the DR region [39, 40]. In this study all the MTBC strains examined had 1 mid DR IS6110 mobile element (Fig. 2). After CRISPR deletion, along with deletion of varying amounts of DR-flanking sequences, the DR region was left with a residual IS6110 mobile element attached to what remained of the flanking sequences (Fig. 2). The IS6110 mobile element has the ability to delete sequences on either side of it [39]. In the MTBC Beijing family, the mid DR IS6110 mobile element is found in reverse orientation to the H37Rv mid DR IS6110 sequence [4, 41]. The results shown in this paper were consistent with these previous findings, as except for the deleted-DR Beijing strains from patient B, all Beijing mid DR IS6110 elements from validated complete chromosome sequences, had orientations inverse (Table 1, and shown in red in Additional File 6, Figure S1) to the mid DR H37Rv IS6110 orientation. In the deleted-DR Beijing strains from patient B, the residual mid DR IS6110 was oriented in the same direction as the reference H37Rv mid DR IS6110 mobile element (Patient B, Additional File 6: Figure S1). We speculate that generation of the deleted-DR extended RD207 deletion involved this IS6110 mobile element, which in the process was “flipped” from its previous characteristic Beijing inverse orientation (presumably generated by formation of the ancestral Beijing-defining RD207 deletion) back to a wild type H37Rv-like orientation. The mechanism behind the IS6110 ability to generate deletions has been investigated [39, 41], but to the best of our knowledge this issue has not been resolved. In summary, it is probable that the residual IS6110 mobile element found attached to the presumed (by homology) DR flanking sequences of the deleted-DR strains (Fig. 2), played an as yet uncharacterized role in the deletion of the CRISPR sequences and flanking genes observed to be deleted in the deleted-DR strains (Fig. 2).

### Validation of total loss of CRISPR spacers in deleted-DR isolates A, B, and C, and in downloaded read-assembled deleted-DR strain SRR1188186

#### Approach 1

Recovery of spoligotypes from NGS data for isolates A, B, and C, and T3_Eth, was accomplished as follows (by LS, Scripps). A bed file was created using the coordinates of the 43 DR repeat units. This bedfile and the aligned bam files were used with “samtools bedcov” tool to output number of counts for each repeat unit. In order to produce a binary table matching the spoligotyping, regions with less than 500 counts were considered absent. By this method, the correct spoligotypes (spacer complement) could be obtained for the intact-DR T3_Eth.

#### Approach 2

In order to validate the total loss of spacers and repeats in the deleted-DR isolates, for each isolate A, B and C corrected DRplus sequences (see above: corrected by substitution of manual PCR-derived contig sequences spanning the DR region) were examined for spacer and repeat sequences, which were found to be totally absent from these corrected DRplus sequences.

#### Approach 3

In order to further confirm the total loss of CRISPR spacers in deleted-DR isolates A, B, and C, and in downloaded read-assembled deleted-DR strain SRR1188186, a new application was developed (by PJF, NPHLTA) for BioNumerics 7.5 and 7.6 and Excel (manuscript in preparation). The application yielded results consistent with other approaches (1 and 2 above) in that no spacers were detected in deleted-DR strains, and correct spoligotypes were assigned to reference H37Rv, reference BCG, and the 3 sequenced T3_Eth.

### CRISPR-Cas defects: comparison of MTBC strains for insertions/deletions and SNPs in *cas* genes

Since isolates from patient B were Beijing, it was of interest that the deleted-DR deletion consisted of a left-side-of-*IS6110* extension (Fig. 2) of the Beijing-defining RD207 deletion. The RD207 deletion is found in all modern Beijing [15, 31], and removes most *CRISPR*-spacers and *cas* genes through Rv2820 (*csm4*) (Fig. 2) [15]. Table 2 lists the *cas* gene mutations in order of ascending location on the H37Rv chromosome (although transcription is in the reverse direction), and the corresponding mutated genes for the strains examined in this study (Table 1). Notable mutations were the extensive deletions of *cas* genes in Beijing family members including deletion of *cas1* (Fig. 2); the deletion of *cas2* and *cas1* by all deleted-DR strains (Fig. 2); T3_Eth specific missense SNPs in *cas1*; and a proto-Beijing specific missense SNP in *cas1* (Table 2, Additional file 7: Figures S4–6). T3_Eth is our largest MDR-containing African-origin cluster, and has been reported by others to have proclivity for MDR [42].

### How likely is it that the Cas1 proteins from Type III-A systems in MTBC also have a role in DNA repair in a manner similar to that of Cas1 proteins from Type I-E in *E. coli*?

Babu et al. 2011 [27] induced DNA damage in *E. coli* by exposure to mitomycin C, UV light or cisplatin. They noted, and gave examples, that Cas1 proteins from different genera of bacteria can display related but different biochemical properties. Thus any given Cas1 protein must be characterized for its biochemical and functional roles, which they did for the E. coli Type I-E Cas1, but which has not yet been done for the MTBC Type III-A Cas1. Moreover, we and others [43] were not able to find any studies done in any genus to determine the biochemical and functional roles of Type III systems. Nevertheless, Makarova et al. 2015 [12] noted that the Cas1 family is the most conserved Cas protein family [44, 45] and showed that the Cas1 phylogenetic tree corresponds nicely with the phylogeny of the bacterial genera. By use of the NCBI tool CDART [46], we observed that the *E. coli* Type I-E Cas1 had homologous domain architecture with the *M. tuberculosis* Type III-A Cas1. The Cas1 proteins from both were members of the Cas1 endonuclease super-family Cas1_I-II-III. Only the *E. coli* Cas1 was labeled “multifunctional” because there is no relevant data yet for *M. tuberculosis* Cas1, however, we consider it likely that the MTBC Cas1 will eventually be shown to have a role in endogenous DNA repair.

It was noted by Makarova et al., 2015, [12] that the adaptation module (cas1 and cas2) is dispensable in subtypes III-A and III-B. This would tend to support the idea that Cas1 in MTBC is nonessential, even if it does have an endogenous DNA-repair activity role. Consistent with this, the Beijing family of MTBC has deleted cas1 and cas2 (Fig. 2), yet still is a very successful pathogen. However, nonessential does not necessarily mean non-important. Cas1 is produced in MTBC [30], except for the possible exceptions noted in our paper (and any other exceptions yet to be found), and its absence or mutation-induced defectiveness could in theory result in impaired DNA repair and consequent increased mutability.

### Has induction of the *cas1* gene been observed in response to DNA-damage in genera other than that of *E. coli* which shows Type I-E *cas1* induction?

*Pyrococcus furiosus* has 7 different CRISPR loci and 3 different CRISPR-Cas systems, Type III-B, Type I-G, and Type I-A [47]. Williams et al., 2007, [48] analyzed the transcriptome of *P. furiosus* after induction of DNA damage by gamma irradiation. They observed 5 genes that had up to a 10-fold increase in mRNA levels following gamma irradiation. 3 of the 5 genes were CRISPR-associated, but none of them was *cas1*, although 1 was predicted to be involved in DNA repair and recombination [48]. Interestingly, all 3 up-regulated CRISPR-associated genes belonged to the Type I-A system [47], and none of the genes in the other 2 systems, Type III-B and Type I-G, were similarly up-regulated [48]. Williams et al., 2007, [48] note that some efficient DNA repair pathways appear to be constitutively expressed in the hyperthermophilic archaeon *P. furiosus*, which they attribute to the result of life at high temperature. To our knowledge there are no studies of induction of *cas1* in response to DNA-damage, other than the report of induction of *cas1* in E. coli [27].

Life in a macrophage, and frequent exposure to hypoxic conditions, also lead to a stressful environment for *M. tuberculosis*, and we ask if this is the reason that Cas1 was found in vitro in the MTBC proteome by Mawyuenyega et al., 2005 [30], that is, is there constitutive expression of the MTBC Type III-A *cas1* gene? Support for this view comes from findings that *cas1* (Rv2817c) is an essential gene for MTBC growth in vitro [49–51]. In contrast, for MTBC H37Rv grown in vivo the *cas1* gene was unessential in an infected-mouse model [52] and the Cas1 protein was not detected in the proteome in an infected-guinea pig model [53]. Perhaps in vivo expression of *cas* genes is regulated differently than in vitro expression. In any case, if *cas1* gene and Cas1 protein are essential for in vitro growth of MTBC, the deleted-DR strains and Beijing family strains (all lacking *cas1*, Fig. 2) and strains with potentially defective *cas1* such as T3_Eth (Table 2, Additional File 7 Figures S2 and S3), must have developed compensatory mechanisms which allowed them to be cultured from clinical samples. Another possibility that has not been examined, is whether *cas1* expression is somehow connected with possible constitutive expression of crRNAs – in *P. furiosus* the approximately 200 crRNAs from all 7 CRISPR loci are constitutively expressed (reviewed in Majumdar et al. 2015 [47]), but to our knowledge nothing is known about the expression of MTBC crRNAs.

### What possible effects might the loss of the *cas1* gene have on any given MTBC strain?

Cas1 protein physically and genetically interacts with key components of *E. coli* DNA repair systems, including *recB*, *recC* and *ruvB*, and *cas1* deleted *E.coli* shows increased sensitivity to DNA damage and impaired chromosomal segregation [27]. Is there the possibility of a similar phenotype in the deleted-DR strains of MTBC, or intact-DR strains of MTBC, that are defective in the *cas1* gene? There is no data with which to answer this question, but some pertinent observations can be made concerning MTBC. As already mentioned, the widespread successful MTBC pathogens of the Beijing family [54, 55] all lack the *cas1* gene as part of their defining RD207 deletion (Fig. 2, Table 2) [15, 31]. Evidence presented in this paper showed that all deleted-DR strains (assigned to Haarlem, Beijing, EAI, and CAS spoligotype families by RD deletion profiles, Table 1) lost at least their *cas1* and *cas2* genes (Fig. 2, Table 2), but previously Beijing was considered the only natural instance known of CRISPR-containing bacteria to be without a *cas1* gene [27]. However this knowledge has been updated by Makarova et al., 2015, [12] who report that the cas1-cas2 adaptation module is dispensable in Types III-A and III-B CRISPR-Cas systems. No evidence has been offered for impaired chromosomal segregation in Beijing MTBC, but there appears to be an increased sensitivity to DNA damage as shown by Beijing-specific SNPs resulting in a potential mutator phenotype (Table 1) [37], and by the fact that in Israel [1] and worldwide [37, 54, 56–58], MTBC Beijing family strains are observed to be robustly transmissible (at least in the patient’s country of origin) and to have an increased tendency to be MDR. Thus one could speculate that in Beijing, loss of *cas1* may have contributed to increased adaptability (caused by increased mutability due to decreased DNA repair function resulting from loss of Cas1 and loss of DNA repair activity from the DNA repair genes/gene products with which Cas1 interacts [27, 59]). In this regard, it is notable that the largest MDR-containing African-origin cluster in Israel [1] and some parts of Africa [42] is T3_Eth, which was shown in this study to have 2 potentially disruptive SNP missense mutations in its cas1 gene (Table 2, Additional file 7, Figures S2 and S3). Further studies are necessary to determine whether T3_Eth has functional Cas1.

MTBC strains differ from most other bacteria by lacking a mismatch repair system [60, 61], the lack of which causes instability within genomic regions containing nucleotide repeats [61]. We previously published a possible instance of this instability observed in a large cluster of CAS family MTBC in Israel [7], and one could speculate that this instability may be one of the drivers of MIRU-VNTR polymorphism in general which is extremely useful in molecular epidemiology of MTBC [5]. However, Wanner et al. [61] analyzed the MTBC strain H37Rv and other bacteria, and determined that deficiency in DNA-repair increases the selection for inherently stable genomes “by using codons to encode proteins in a context-dependent manner that prevents the emergence of nucleotide repeats.” The results reported and summarized in our paper suggest that different families of MTBC have different burdens of DNA-repair deficiency (Table 1, Table 2), and thus the important observation of stabilization of the H37Rv genome may not be generalizable to all MTBC without examining other strains belonging to at least the other major families of MTBC.

The effect of defective DNA-repair genes is cumulative: the more defective DNA-repair genes, the more mutability [62], which presumably could lead to increased adaptability, with consequent accumulation of mutations in global transcription/stress response genes favoring survivability/persistence, transmissibility, and acquisition of drug resistance. All MTBC strains, as noted above, lack the mismatch repair system, and it is therefore important to draw attention to the possible cumulative effect in Beijing set in motion by the additional loss of *cas1* (Fig. 2, Table 2) and its putative associated DNA-repair network [27], in addition to other known Beijing-specific defective DNA-repair genes, (Table 1, [37]). The DNA-repair mechanism in MTBC is already known to be one of the slowest in bacteria [59], and cumulative loss of DNA-repair functionality could only be expected to make the DNA-repair mechanism even slower, exacerbating the acquisition of mutations known to result from defective DNA-repair [62].

## Conclusions

Deleted-DR strains showed complete loss of clustered regularly interspaced short palindromic repeats (CRISPR) and accompanying spacers, retention of the mid-CRISPR IS6110 mobile element in clonally specific orientation, and accompanying clonally-specific loss of flanking genes, including adjacent CRISPR-associated (*cas*) genes [12]. All deleted-DR strains were accompanied by a loss of at least the *cas2* and *cas1* genes. Tentative MIRU-VNTR-based assignments of the deleted-DR isolates to MTBC major spoligotype families A Haarlem, B Beijing, and C EAI were validated by identifying lineage-defining long sequence polymorphisms (LSPs) known as region of difference (RD) deletions [14, 15, 17], corresponding to major spoligotype families [17]. Similarly, the deleted-DR strain SRR1188186 whose sequence was assembled from downloaded reads, deleted *cas2* and *cas1* and was assigned by RD deletion to the MTBC major spoligotype family CAS. Since *cas1* deleted *E.coli* shows increased sensitivity to DNA damage and impaired chromosomal segregation [27], we discussed the possibility of a similar phenotype in the deleted-DR strains of MTBC and in the intact-DR MTBC Beijing that are defective in the *cas1* gene (having deleted it), and in the intact-DR MTBC T3_Eth that are potentially defective in the *cas1* gene (having 2 potentially disruptive missense SNPs in it).

## Additional files

Additional file 1: Table S1. MIRU-VNTR loci and octal spoligotypes. Samples: A-B-C&T3_Eth. Controls: 0486–12 Beijing clinical isolate, ECDCPT 21&25 EAI, reference H37Rv, Pasteur BCGP2. (DOCX 19 kb)
Additional file 2:
**List S1**. Sources for drug sensitivity data presented in Table 1. (DOCX 15 kb)
Additional file 3:
**Sequences S1–S6. **(ZIP 66 kb)
Additional file 4: Table S2. Location of CRISPR-flanking genes and mid-CRISPR IS6110 mobile element used for primer choices and alignments. (DOCX 17 kb)
Additional file 5: Table S3. Primers employed for PCR verification of deletions of CRISPR/DR and flanking regions. (DOCX 14 kb)
Additional file 6: Figure S1. Visualization of mid IS6110 orientation: blue for wild type (as in reference H37Rv) or for unknown orientation; red for inverse orientation (see Table 1). DR plus flanking sequences were aligned to DR and flanking regions of reference template H37Rv. Sequences were from *Mycobacterium tuberculosis* NGS read sets generated in this study (patients A, B, and C corrected by manual PCR; and T3_Eth), on-line resources of read sets, mapped on H37Rv NC_000962.3, and on-line resources of complete chromosome sequences. (PPT 481 kb)
Additional file 7: Figures S2–S4. Mutations of *cas1* shared by MTBC T3_Eth strains and by Proto-Beijing strains. (PPTX 2956 kb)

## Abbreviations

Cas: CRISPR-associated
CAS: Central Asian (refers to a major spoligotype and corresponding RD deletion family)
*cas*: CRISPR-associated gene
CRISPR: Clustered regularly interspaced short palindromic repeats
DR: Direct repeat region
DRplus: Sequence of DR plus flanking regions – about 40 Kbp
LSP: Long sequence polymorphism
MIRU-VNTR: Mycobacterial interspersed repetitive units – variable number tandem repeats
MTBC: *Mycobacterium* *tuberculosis* complex
NGS: Next generation sequencing
RD: Region of difference
spoligotyping: Spacer oligonucleotide typing
