# Supplementary material for: Structure and variation of CRISPR and CRISPR-flanking regions in deleted-direct repeat region Mycobacterium tuberculosis complex strains
Source: BMC Genomics. 2017 Feb 15;18:168. doi: 10.1186/s12864-017-3560-6 (PMC5310062; doi:10.1186/s12864-017-3560-6)
Supplement: Additional file 1: Table S1. — MIRU-VNTR loci and octal spoligotypes. Samples: A-B-C&T3_Eth. Controls: 0486–12 Beijing clinical isolate, ECDCPT 21&25 EAI, reference H37Rv, Pasteur BCGP2. (DOCX 19 kb) [file 12864_2017_3560_MOESM1_ESM.docx]

| **Additional file 1: Table S1.** MIRU-VNTR loci and octal spoligotypes. Samples: A-B-C&T3_Eth.  Controls: 0486-12 Beijing clinical isolate, ECDCPT 21&25 EAI, reference H37Rv, Pasteur BCGP2. |  |  |
| --- | --- | --- |
| **MIRU-VNTR Locus**  02 04 10 16 20 23 24 26 27 31 39 40 A B C M M M M M M Q Q Q | |  |

04 21 29 30 34 39 11b 4052 4156c

**ID* Copy numbers**

| Octal spoligotype 000000000000000  A_0389-12 2 2 3 3 2 5 1 5 3 3 2 3 3 2 3 2 3 4 4 3 3 2 5 3  A­_1124-11 2 2 3 3 2 5 1 5 3 3 2 3 3 2 3 2 3 4 4 3 3 2 5 3  B_1042-11 2 2 3 3 2 5 1 7 3 5 5 3 4 2 4 5 6 4 4 3 3 5 8 2  B_1114-11 2 2 3 3 2 5 1 7 3 5 5 3 4 2 4 5 6 4 4 3 3 5 8 2  C_0253-12 2 5 4 3 3 6 2 2 3 4 3 2 4 6 4 1 10 3 2 3 2 9 7 1  C_0309-12 2 5 4 3 3 6 2 2 3 4 3 2 4 6 4 1 10 3 2 3 2 9 7 1  Octal spoligotype 777000377760771  T3_Eth_0140-11 2 2 5 1 2 5 1 1 3 3 2 2 3 2 4 1 3 4 4 3 3 1 7 3  T3_Eth_0192-11 2 2 5 1 2 5 1 1 3 3 2 2 3 2 4 1 3 4 5 3 3 1 7 3  T3_Eth_0525-11 2 2 5 1 2 5 1 1 3 3 2 2 3 2 4 1 2 4 4 3 3 1 7 3  Octal spoligotype 000000000003771  0486-12 2 2 3 3 2 5 1 7 3 5 3 3 4 2 4 4 5 4 4 3 3 6 7 2 |
| --- |
| Octal spoligotype 677777477413771  ECDCPT21 2 5 4 3 3 6 2 2 3 4 3 2 4 6 4 1 10 3 2 3 2 9 7 1  ECDCPT25 2 5 4 3 3 6 2 2 3 4 3 2 4 6 4 1 10 3 2 3 2 9 7 1  Octal spoligotype 777777477760771  H37Rv 2 3` 3 2 2 6 1 3 3 3 2 1 3 3 4 2 2 4 2 3 5 5 5 2  Octal spoligotype 676773777777600  BCGP2 2 2` 2 3 2 4 2 5 3 3 2 2 5 5 6 0 1 2 2 3 2 3 5 0 |

*ID, identification; ECDCPT21,25 European Center for Disease Control Proficiency Test 2013 sample numbers 21,25 and control H37Rv (deBeer et al., RIVM); BCGP2, Pasteur BCG strain P2, a kind gift from Hillel Bercovier, Hebrew University of Jerusalem;

SIT family, spoligotype international type family: 0140-11, 0192-11, 0525-11: **T3_Eth** SIT149 from largest National Mycobacterium Reference Center Tel-Aviv (NMRC) MDR-containing African-origin cluster; 0486-12, **Beijing** SIT1 from large Beijing cluster of NMRC, (SIT number and family from Brudey et al., SpolDB4, 2006, http://www.pasteur-guadeloupe.fr/tb/bd_myco.html, or in case of all zeros spoligotype, the family frequently associated with the given MIRU-VNTR lineage plus defining RD deletion)—A_0389-12, A_1124-11, **Haarlem**: Kremer et al., 1999, and deleted RD182 Table 2 (Coll et al., 2014); B_1042-11, B_1114-11, **Beijing**: Kam et al., 2005, Gagneux et al., 2006 – share Beijing-defining RD105 deletion and Beijing-specific mutator gene SNPs (Table 2); **EAI Manilla**: identity of MIRU-VNTR profiles to those of ECDCPT strains 21 and 25 (deBeer et al., RIVM) with **EAI** **Manilla** spoligotypes spolDB4 SIT19, and deleted RD239 Table 2 (Coll et al., 2014).
