# Supplementary material for: Structure and variation of CRISPR and CRISPR-flanking regions in deleted-direct repeat region Mycobacterium tuberculosis complex strains
Source: BMC Genomics. 2017 Feb 15;18:168. doi: 10.1186/s12864-017-3560-6 (PMC5310062; doi:10.1186/s12864-017-3560-6)
Supplement: Additional file 2: — List S1. Sources for drug sensitivity data presented in Table 1. (DOCX 15 kb) [file 12864_2017_3560_MOESM2_ESM.docx]

**Additional file 2: List S1.** Sources for drug sensitivity data presented in Table 1.

^a^ Tsui,SKW. Genome analysis of Mycobacterium tuberculosis Beijing family strains. Hong Kong Med J 2013; 19:12-14.

^b^ Zhang,Y et al. Complete genome sequences of Mycobacterium tuberculosis strains CCDC5079 and CCDC5080, which belong to the Beijing family. Journal of Bacteriology 2011; 193:5591-5592.

^c^ Rodriguez, JG et al. Complete genome sequence of the clinical Beijing-like strain Mycobacterium tuberculosis 323 using the PacBio real-time sequencing platform. genomeA 2015; 3:1-2.

^d^ Coll, F et al. A robust SNP barcode for typing Mycobacterium tuberculosis complex strains. Nat. Commun. 2014; 5:4812. [used TB profiler

^e^ this paper

^f^ BCG is by definition resistant to pyrazinamide

^g^ Ilina EN et al. Comparative genomic analysis of Mycobacterium tuberculosis drug resistant strains from Russia. PLoS ONE 2013; 8:e56577.
