# Supplementary material for: Structure and variation of CRISPR and CRISPR-flanking regions in deleted-direct repeat region Mycobacterium tuberculosis complex strains
Source: BMC Genomics. 2017 Feb 15;18:168. doi: 10.1186/s12864-017-3560-6 (PMC5310062; doi:10.1186/s12864-017-3560-6)
Supplement: Additional file 4: Table S2. — Location of CRISPR-flanking genes and mid-CRISPR IS6110 mobile element used for primer choices and alignments. (DOCX 17 kb) [file 12864_2017_3560_MOESM4_ESM.docx]

**Additional file 4: Table S2.** Location of CRISPR-flanking genes and mid-CRISPR IS6110 mobile element used for primer choices and alignments

| **Gene** | **Protein** | **Starts (H37Rv)** | **Ends (H37Rv)** | **Length (bp)** | **B** | **CRISPR (DR) & Flanking Regions**  **Del-DR Strains**  **C** | **A** |
| --- | --- | --- | --- | --- | --- | --- | --- |
|  |  |  |  |  | **1042-11**  **1114-11** | **0253-12**  **0309-12** | **0389-12**  **1124-11** |
| Rv2809 | NP_217325.1 | 3115408 | 3115719 | 258 | pd | p | p |
| IS1555 | Insertion element | 3115744 | 3116142 | 399 | d | d | p |
| Rv2811 | NP_217327 | 3116139 | 3116747 | 609+1 | d | d | p |
| Rv2812 | NP_217328.1 | 3116818 | 3118227 | 1410+1 | d | d | p |
| Rv2813 | NP_217329.1 | 3118224 | 3119036 | 813+1 | d | d | p |
| DR region 1 |  | 3119185 | 3120523 | 1338 | d | d | d |
| IS6110 |  | 3120523 | 3121897 | 1375 | p | p | p |
| DR region 2 |  | 3121882 | 3123576 | 1694 | d | d | d |
| Rv2816c | NP_317332.1 | 3123625 | 3123966 | 342 | d | d | d |
| Rv2817c | NP_217333.1 | 3123967 | 3124983 | 1017 | d | d | d |
| Rv2818c | NP_317334.1 | 3124996 | 3126144 | 1149 | d | p | pd |
| Rv2819c | NP_217335.1 | 3126240 | 3127367 | 1128 | d | p | p |
| Rv2820c | NP_217336.1 | 3127364 | 3128272 | 909 | pd | p | p |
| Rv2821c | NP_217337.1 | 3128253 | 3128963 | 711 | p | p | p |
|  |  |  |  |  |  |  |  |
| **d – deleted, pd – partially deleted, p – present** | | | | |  |  |  |
