# Supplementary material for: Structure and variation of CRISPR and CRISPR-flanking regions in deleted-direct repeat region Mycobacterium tuberculosis complex strains
Source: BMC Genomics. 2017 Feb 15;18:168. doi: 10.1186/s12864-017-3560-6 (PMC5310062; doi:10.1186/s12864-017-3560-6)
Supplement: Additional file 5: Table S3. — Primers employed for PCR verification of deletions of CRISPR/DR and flanking regions. (DOCX 14 kb) [file 12864_2017_3560_MOESM5_ESM.docx]

**Additional file 5: Table S3.** Primers employed for PCR verification of deletions of CRISPR/DR and flanking regions.

| No. | Primer name | Sequence | 5' position in H37Rv |
| --- | --- | --- | --- |
| 1 | 3114969F | AGACGCAACAGTGGTCCTAA | 3114968 |
| 2 | 3114688F | ATCAACGCGATCCAAATGCA | 3114687 |
| 3 | 3125062R | TTGACGCTCTATGACCGGTT | 3125061 |
| 4 | 3125691R | CCAACCTGAAGCAGCTGATC | 3125690 |
| 5 | 3128274R | CATGAACTCGCGGCTGTTTA | 3128273 |
| 6 | 3128515R | CGCGTTCTCACTTGTCTACG | 3128514 |
| 7 | 3114969matchR | CGGCCACGTCTCTAAGTATG | 3115291 |
| 8 | 3128515matchF | CGCCAGCTTCTTCTGCATAC | 3127997 |
| 9 | 3119095F | TTCATGACCAAACGTCCTCA | 3119096 |
| 10 | 3120778R | AATGCACTAGCCGAGACGAT | 3120778 |
| 11 | 3120914R | AGAAGGCGTACTCGACCTGA | 3120914 |
| 12 | 3121331R | CGAACTCAAGGAGCACATCA | 3121331 |
| 13 | 3115545F | ACCGATAATCGCTTGACACC | 3115296 |
| 14 | 309_253_R | AACCTGAAGCAGCTGATCGT | 3125688 |
| 15 | 309_253_R2 | GAGGCTGGAACACCTCGTAG | 3125575 |
| 16 | 3097277_F | TGCGGCTCTTGACAAATCAC | 3118657 |
| 17 | 3097551_F | CAACAACCTCGCCCTGCAAG | 3118931 |
| 18 | 3099600_R | CACCGCGTTCACGTATGACC | 3125536 |
