# Supplementary material for: Structure and variation of CRISPR and CRISPR-flanking regions in deleted-direct repeat region Mycobacterium tuberculosis complex strains
Source: BMC Genomics. 2017 Feb 15;18:168. doi: 10.1186/s12864-017-3560-6 (PMC5310062; doi:10.1186/s12864-017-3560-6)
Supplement: Additional file 6: Figure S1. — Visualization of mid IS6110 orientation: blue for wild type (as in reference H37Rv) or for unknown orientation; red for inverse orientation (see Table 1). DR plus flanking sequences were aligned to DR and flanking regions of reference template H37Rv. Sequences were from Mycobacterium tuberculosis NGS read sets generated in this study (patients A, B, and C corrected by manual PCR; and T3_Eth), on-line resources of read sets, mapped on H37Rv NC_000962.3, and on-line resources of complete chromosome sequences. (PPT 481 kb) [file 12864_2017_3560_MOESM6_ESM.ppt]

## Slide 1
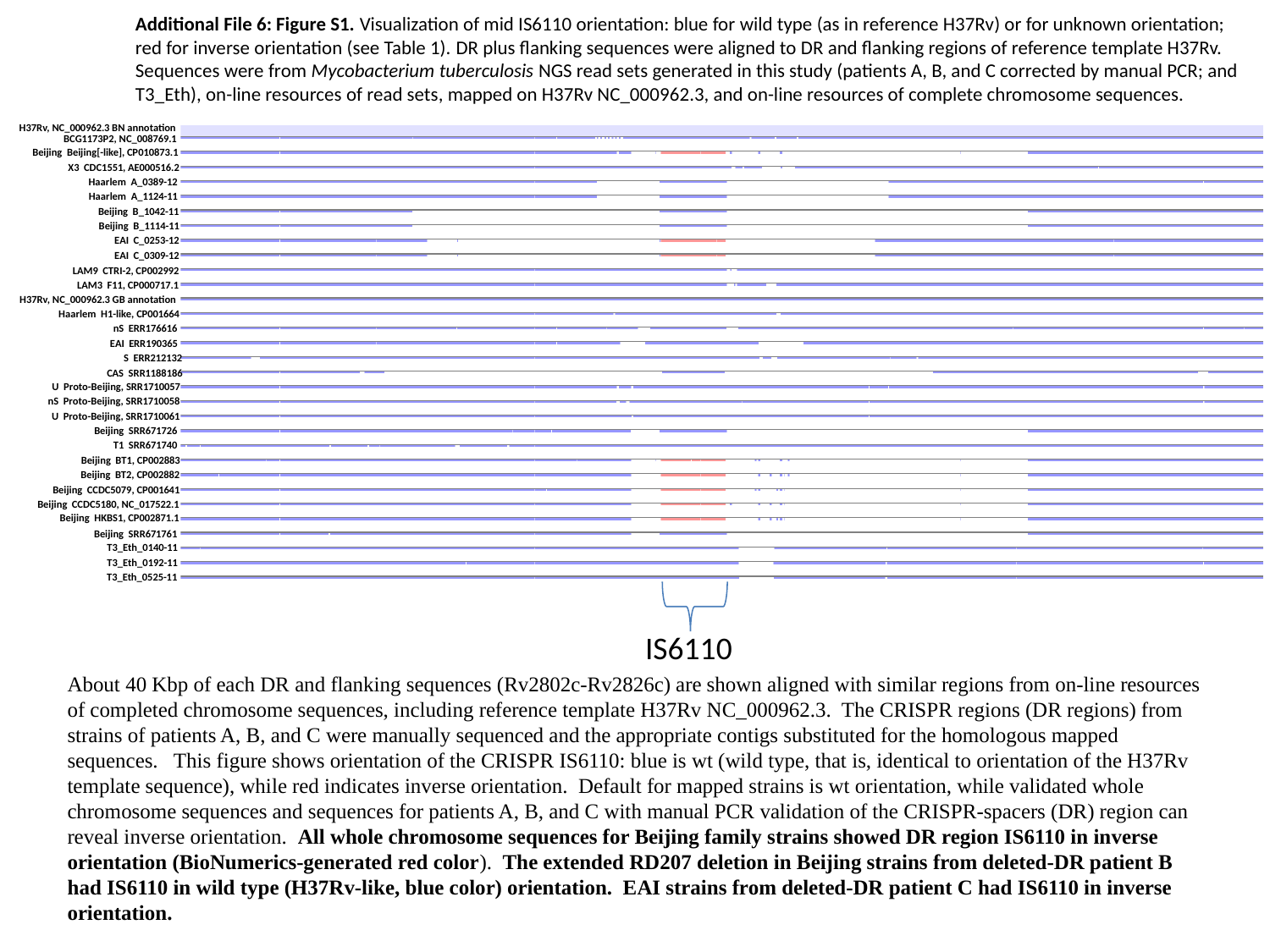

Additional File 6: Figure S1. Visualization of mid IS6110 orientation: blue for wild type (as in reference H37Rv) or for unknown orientation; red for inverse orientation (see Table 1). DR plus flanking sequences were aligned to DR and flanking regions of reference template H37Rv. Sequences were from Mycobacterium tuberculosis NGS read sets generated in this study (patients A, B, and C corrected by manual PCR; and T3_Eth), on-line resources of read sets, mapped on H37Rv NC_000962.3, and on-line resources of complete chromosome sequences.
H37Rv, NC_000962.3 BN annotation
BCG1173P2, NC_008769.1
Beijing Beijing[-like], CP010873.1
X3 CDC1551, AE000516.2
Haarlem A_0389-12
Haarlem A_1124-11
Beijing B_1042-11
Beijing B_1114-11
EAI C_0253-12
EAI C_0309-12
LAM9 CTRI-2, CP002992
LAM3 F11, CP000717.1
H37Rv, NC_000962.3 GB annotation
Haarlem H1-like, CP001664
nS ERR176616
EAI ERR190365
S ERR212132
CAS SRR1188186
U Proto-Beijing, SRR1710057
nS Proto-Beijing, SRR1710058
U Proto-Beijing, SRR1710061
Beijing SRR671726
T1 SRR671740
Beijing BT1, CP002883
Beijing BT2, CP002882
Beijing CCDC5079, CP001641
Beijing CCDC5180, NC_017522.1
Beijing HKBS1, CP002871.1
Beijing SRR671761
T3_Eth_0140-11
T3_Eth_0192-11
T3_Eth_0525-11
IS6110
About 40 Kbp of each DR and flanking sequences (Rv2802c-Rv2826c) are shown aligned with similar regions from on-line resources of completed chromosome sequences, including reference template H37Rv NC_000962.3. The CRISPR regions (DR regions) from strains of patients A, B, and C were manually sequenced and the appropriate contigs substituted for the homologous mapped sequences. This figure shows orientation of the CRISPR IS6110: blue is wt (wild type, that is, identical to orientation of the H37Rv template sequence), while red indicates inverse orientation. Default for mapped strains is wt orientation, while validated whole chromosome sequences and sequences for patients A, B, and C with manual PCR validation of the CRISPR-spacers (DR) region can reveal inverse orientation. All whole chromosome sequences for Beijing family strains showed DR region IS6110 in inverse orientation (BioNumerics-generated red color). The extended RD207 deletion in Beijing strains from deleted-DR patient B had IS6110 in wild type (H37Rv-like, blue color) orientation. EAI strains from deleted-DR patient C had IS6110 in inverse orientation.
