# Supplementary material for: Structure and variation of CRISPR and CRISPR-flanking regions in deleted-direct repeat region Mycobacterium tuberculosis complex strains
Source: BMC Genomics. 2017 Feb 15;18:168. doi: 10.1186/s12864-017-3560-6 (PMC5310062; doi:10.1186/s12864-017-3560-6)
Supplement: Additional file 7: Figures S2–S4. — Mutations of cas1 shared by MTBC T3_Eth strains and by Proto-Beijing strains. (PPTX 2956 kb) [file 12864_2017_3560_MOESM7_ESM.pptx]

## Slide 1
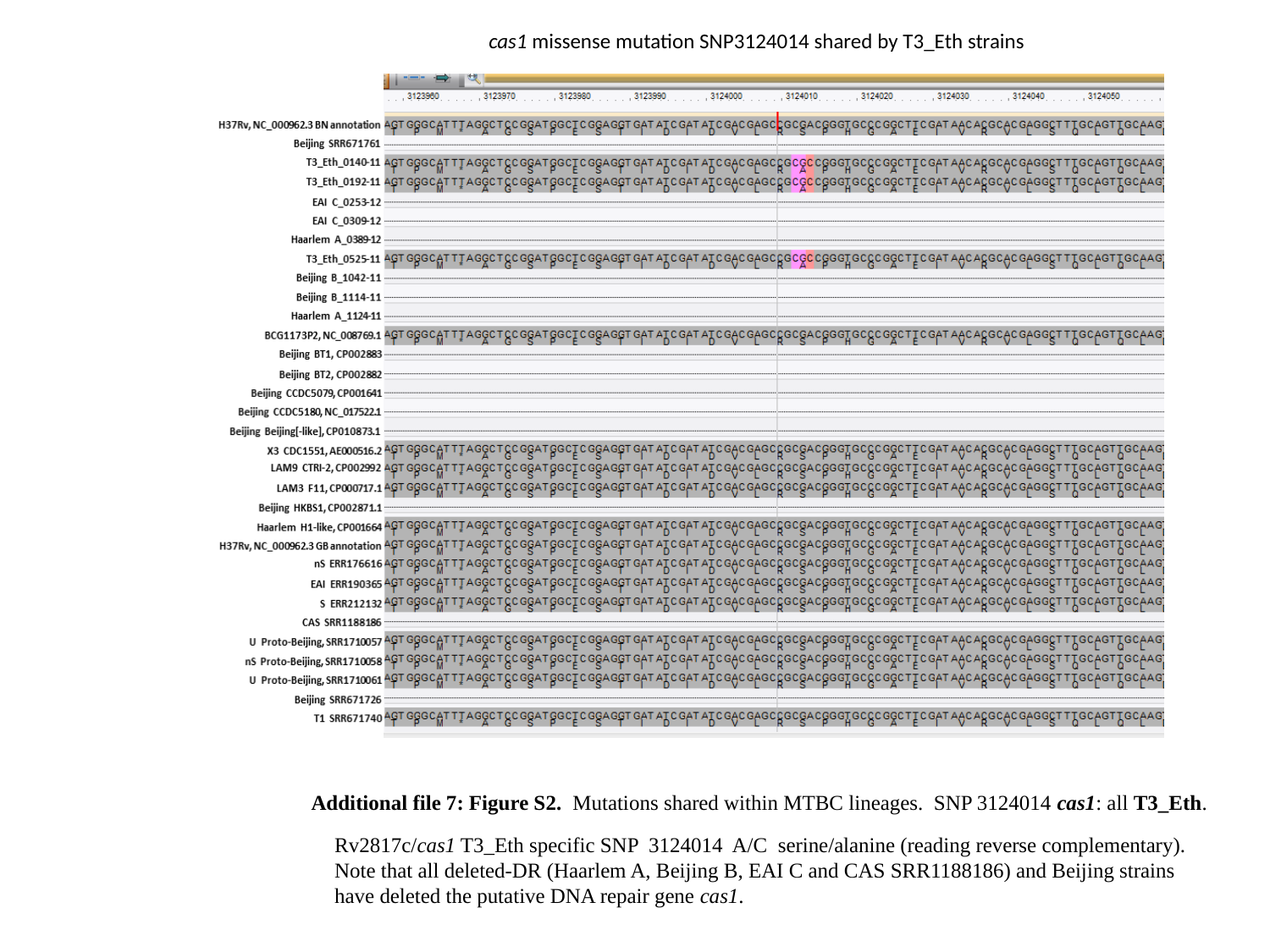

cas1 missense mutation SNP3124014 shared by T3_Eth strains
Additional file 7: Figure S2. Mutations shared within MTBC lineages. SNP 3124014 cas1: all T3_Eth.
Rv2817c/cas1 T3_Eth specific SNP 3124014 A/C serine/alanine (reading reverse complementary). Note that all deleted-DR (Haarlem A, Beijing B, EAI C and CAS SRR1188186) and Beijing strains have deleted the putative DNA repair gene cas1.

## Slide 2
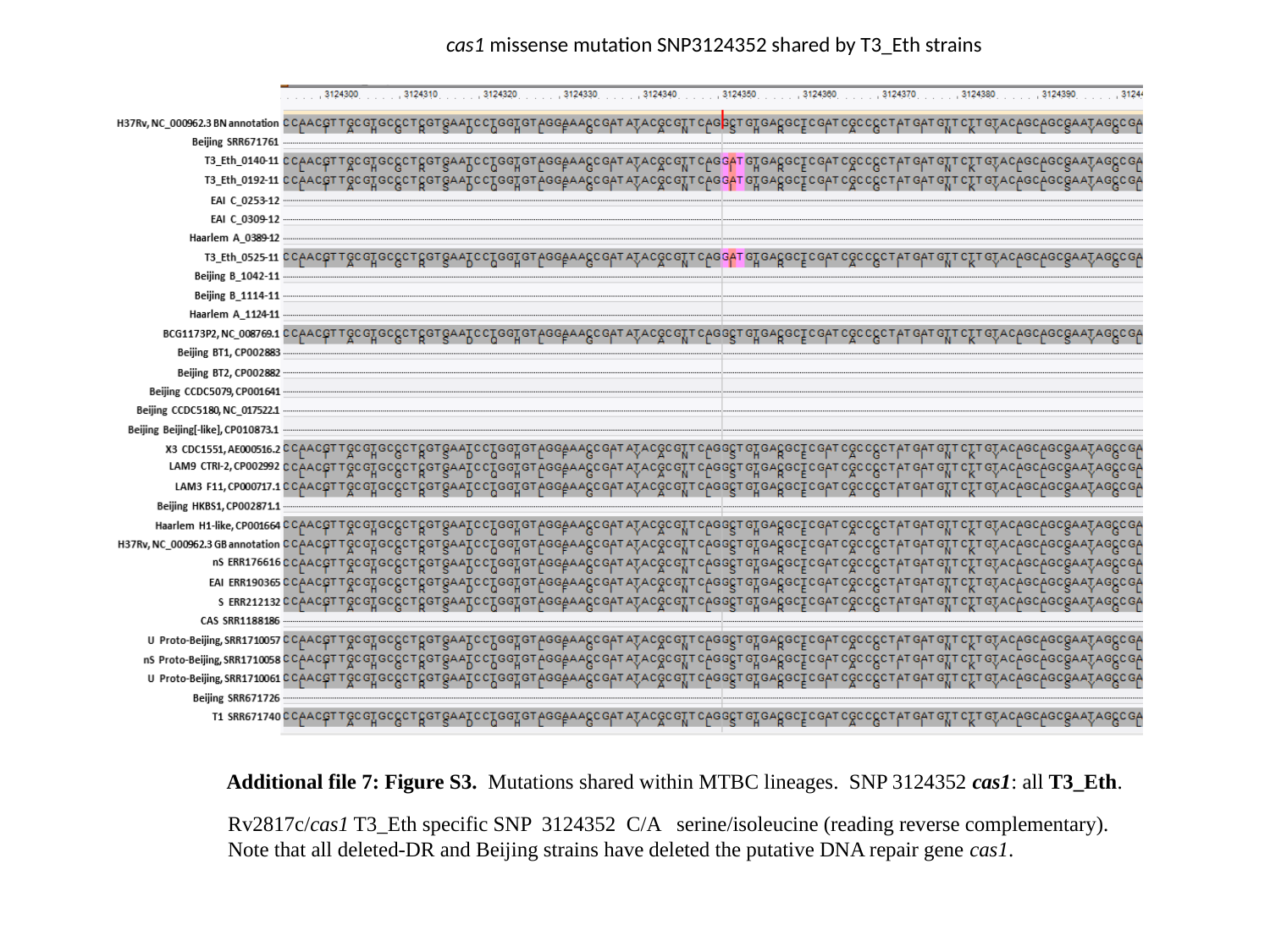

cas1 missense mutation SNP3124352 shared by T3_Eth strains
Additional file 7: Figure S3. Mutations shared within MTBC lineages. SNP 3124352 cas1: all T3_Eth.
Rv2817c/cas1 T3_Eth specific SNP 3124352 C/A serine/isoleucine (reading reverse complementary). Note that all deleted-DR and Beijing strains have deleted the putative DNA repair gene cas1.

## Slide 3
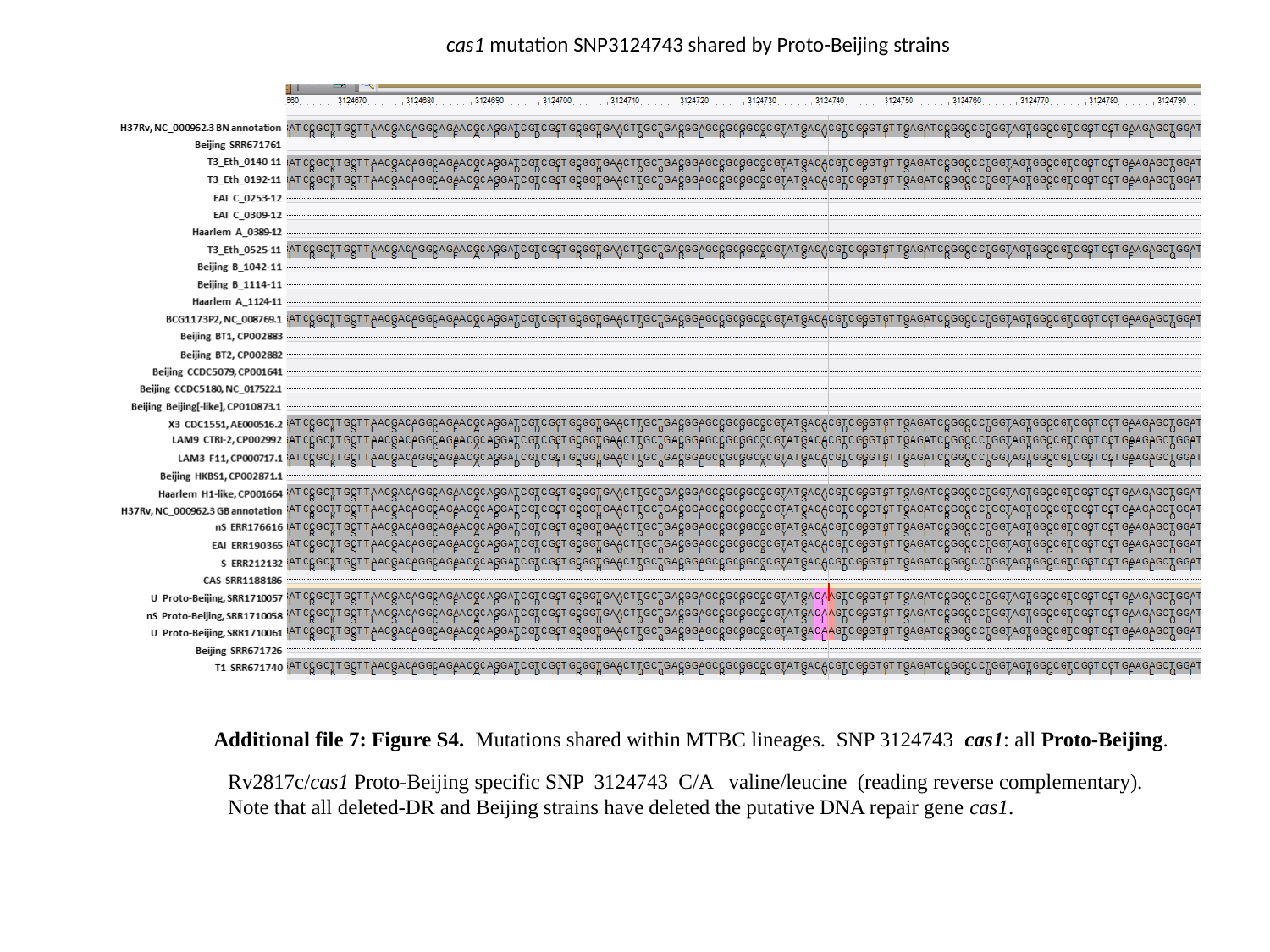

cas1 mutation SNP3124743 shared by Proto-Beijing strains
Additional file 7: Figure S4. Mutations shared within MTBC lineages. SNP 3124743 cas1: all Proto-Beijing.
Rv2817c/cas1 Proto-Beijing specific SNP 3124743 C/A valine/leucine (reading reverse complementary). Note that all deleted-DR and Beijing strains have deleted the putative DNA repair gene cas1.
